# Nitric Oxide Synthases in Rheumatoid Arthritis

**DOI:** 10.3390/molecules28114414

**Published:** 2023-05-29

**Authors:** Jia-Bao Huang, Zhi-Ru Chen, Shu-Long Yang, Fen-Fang Hong

**Affiliations:** 1 Experimental Center of Pathogen Biology, Nanchang University, Nanchang 330031, China; j.huang@se19.qmul.ac.uk (J.-B.H.); zhiru.chen@se19.qmul.ac.uk (Z.-R.C.); 2 Queen Mary School, Nanchang University, Nanchang 330006, China; 3 School of Basic Medical Sciences, Fuzhou Medical College of Nanchang University, Fuzhou 344000, China; 4 Key Laboratory of Chronic Diseases, Fuzhou Medical University, Fuzhou 344000, China; 5 Technology Innovation Center of Chronic Disease Research in Fuzhou City, Fuzhou Science and Technology Bureau, Fuzhou 344000, China

**Keywords:** nitric oxide synthases, nitric oxide, rheumatoid arthritis, inflammatory cytokines, disease-modifying antirheumatic drugs

## Abstract

Rheumatoid arthritis (RA) is an autoimmune disease characterized by severe joint damage and disability. However, the specific mechanism of RA has not been thoroughly clarified over the past decade. Nitric oxide (NO), a kind of gas messenger molecule with many molecular targets, is demonstrated to have significant roles in histopathology and homeostasis. Three nitric oxide synthases (NOS) are related to producing NO and regulating the generation of NO. Based on the latest studies, NOS/NO signaling pathways play a key role in the pathogenesis of RA. Overproduction of NO can induce the generation and release of inflammatory cytokines and act as free radical gas to accumulate and trigger oxidative stress, which can involve in the pathogenesis of RA. Therefore, targeting NOS and its upstream and downstream signaling pathways may be an effective approach to managing RA. This review clearly summarizes the NOS/NO signaling pathway, the pathological changes of RA, the involvement of NOS/NO in RA pathogenesis and the conventional and novel drugs based on NOS/NO signaling pathways that are still in clinical trials and have good therapeutic potential in recent years, with an aim to provide a theoretical basis for further exploration of the role of NOS/NO in the pathogenesis, prevention and treatment of RA.

## 1. Introduction

Rheumatoid arthritis (RA), a chronic autoimmune disease, is characterized by irreversible cartilage and bone damage as well as processive disability, systemic complication, early death and socioeconomic costs. The basic pathological changes are chronic synovitis, pannus formation, and the progressive development of articular cartilage and bone damage. The incidence of RA is 0.5–1% and decreases significantly from north to south and from urban to rural areas in the northern hemisphere [1]. Women are affected two to three times more often than men and incidence of new RA diagnoses peaks in the sixth decade of life. It is two to three times more prevalent in those with a family history of RA than those without, and the prevalence in twins is highly consistent, suggesting a genetic component to the cause of RA. In clinical practice, the manifestation of RA indicates as a symmetrical disease, initially affecting joints from small to larger [2]. It not only primarily involves the joints, such as synovial inflammation and hyperplasia, but also includes extra-articular symptoms, such as cardiovascular, pulmonary, skeletal and psychological disorders [3]. Recent papers have reported that meningitis, brain vasculitis and autoimmune encephalitis had been observed in RA patients [4], which can cause severe cognitive decline, such as in short term memory, immediate and delayed episodic recall and phonemic fluency [3]. RA is a chronic disease that causes musculoskeletal deformities, decreased physical function and cumulative complications that can lead to a subsequent decline in quality of life, which can be a sinking blow to individuals and families.

Until now, the cause of RA remains unknown and effective treatment for RA has not been found. However, nitric oxide synthase (NOS)/Nitric oxide (NO) pathway has been proven to have an important role in the process of pathogenesis of RA and its complications [5]. NO, a kind of gas messenger molecule with many molecular targets and plays important roles in several systems, is produced by NOS [5]. Some studies indicate that the concentration of NO is higher in synovial fluid and blood of RA patients than normal people [6,7,8,9]. Moreover, the concentration of NO in synovial fluid in the active stage is higher than in blood in RA patients. Overproduction of NO is closely related with the generation and release of inflammatory cytokines, and it acts as free radical gas which accumulates and triggers oxidative stress [10], which can be involved in the pathogenesis of RA. Therefore, targeting NO/NOS pathways may be a viable and effective therapeutic approach and there is an urgent requirement to discover and develop novel, natural, safe, and effective anti-arthritis antioxidant and anti-inflammatory compounds as candidates for therapies. This review clearly summarizes the NOS/NO signaling pathways, the pathological changes of RA, the involvement of NOS/NO in RA pathogenesis and conventional and novel drugs based on NOS/NO signaling pathways in recent years, and aims to provide a theoretical basis for further exploration of the role of NOS/NO in the pathogenesis, prevention and treatment of RA.

## 2. NO/NOS Pathway Overview

NO, a small gas molecule, is considered to play significant roles in the cardiovascular, immune and nervous systems and can be generated in the cytosol, the mitochondrial membrane and at the immunological synapse of T cells. NO can diffuse freely in membranes so that it can act as message molecule to mediate various biological functions, such as regulating systolic and diastolic functions of blood vessels, T cells signaling pathways, inflammation, oxidative stress, mitochondrial functions and apoptosis. It mediates signal transduction by regulating Ca^2+^ signaling pathways, by changing the formation of immunological synapses, or through the modification of intra-cellular proteins.

**Figure 1:**
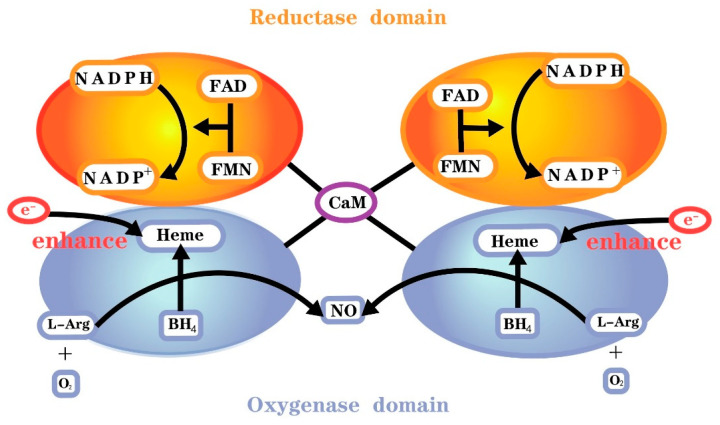
Functional nitric oxide synthase structure.

NOS is associated with generation of NO, which can transform L-arginine to L-citrulline and then produce NO. NOS include three isoforms encoded by different genes, neuronal nitric oxide synthase (eNOS), inducible nitric oxide synthase (iNOS) and endothelial nitric oxide synthase (eNOS) [11]. Molecular oxygen, reduced nicotinamide adenine dinucleotide phosphate (NADPH) and L-arginine serve as the substrate and co-substrates, respectively, for all isoforms of NOS [11]. All isoenzymes require the cofactors flavin adenine dinucleotide (FAD), flavin mononucleotide (FMN) and 6R-l-erythro-5,6,7,8-tetrahydrobiopterin (BH_4_). The reductase domain of this monomer consists of NADPH, FMN and FAD binding sites, whereas the oxygenase domain consists of BH_4_, O_2_ and L-arginine binding sites and a heme group for NOS dimerization (Figure 1). There is also a calmodulin binding domain located between the reductase and oxygenase domains [12].

The first step of NO synthesis is the transfer of electrons from reduced NADPH to FAD and FMN by NOS monomers and the reduction of molecular oxygen to superoxide (O_2_**^−^**) [13]. However, the reductase domain separated by the two monomers needs to be bound to calmodulin (CaM) to enhance electron transport in the reductase domain. All isoforms of NOS bind calmodulin, hence presenting catalytic activity, but the calmodulin binding domains of NOS differ among isoforms. Calmodulin binding in nNOS and eNOS requires elevated Ca^2+^, and calmodulin binds iNOS with high affinity even in the absence of Ca^2+^ [14]. The second step is binding to the cofactor BH_4_ or the substrate L-arginine. The two NOS monomers need to form a complete functional dimer in the presence of haem to catalyze NO formation. Additionally, heme is required for interdomain electron transfer from flavin to heme of the opposite monomer [11]. First, NOS hydroxylates L-arginine to NV-hydroxy-L-arginine. Flavin then transfers an electron from NADPH in the reductase domain of one monomer to heme in the oxygenase domain of the other monomer. By reducing and activating O_2_ at the heme site, N-Ω-hydroxy-L-arginine is converted to L-citrulline and NO [15].

nNOS is expressed in the brain, spinal cord, sympathetic ganglia, skeletal muscle, epithelial cells and vascular smooth muscle. The enzyme activity is modulated by calcium and calmodulin. Different forms of nNOS exists in in different cells with distinct functions. In the central nervous system, nNOS is involved in regulating physiological functions such as memory, learning, neurogenesis and mediating long-term regulation of synaptic transmission. In addition, NO formed by nNOS in the central nervous system is involved in the central regulation of blood pressure [16]. In the periphery, many smooth muscle tissues are innervated by nitro-energic nerves, which are nerves that contain nNOS and produce and release NO. NO produced by nNOS in nitroergic nerves can be regarded as an unusual neurotransmitter that stimulates NO-sensitive guanylate cyclase in its target cells, thereby reducing the tension of various types of smooth muscle, including blood vessels, leading to vasodilation independent of eNOS [17]. 

eNOS is mostly expressed in endothelial cells, while it is also found in certain brain neurons, platelets, cardiomyocytes and renal tubular epithelial cells. Similar to nNOS, Ca^2+^ activated calmodulin is crucial for controlling the activity of eNOS [18]. Additionally, a number of other proteins engage in interactions with eNOS and control its activity. For instance, heat shock protein 90 (HSP90) functions as an allosteric regulator that encourages eNOS coupling, as well as as an eNOS-related activator. The Caveolae coat protein Caveolin-1 is a tonic inhibitor of eNOS activity, which can interact with the eNOS fraction. Recruitment of calmodulin and HSP90 to eNOS dislocates caveolin-1 from the enzyme, resulting in enzyme activation [19]. However, stimulation that does not result in a long-lasting rise in intracellular Ca^2+^ can trigger another independent method of eNOS activation. Multiple serine (Ser), threonine (Thr) and tyrosine (Tyr) residues in the eNOS protein can phosphorylate to raise the Ca^2+^ sensitivity enzyme and eNOS activity [14]. Persistent NO release was induced in the presence of Ca^2+^ deficiency. Numerous crucial cardiovascular processes are homeostatically regulated by eNOS. All kinds of blood arteries are dilated by endothelial NOS-derived NO, which does so via activating soluble guanylate cyclase and raising smooth muscle cell cGMP. Nitric oxide can reduce the expression of chemotactic protein monocyte chemoattractant protein-1 and inhibit leukocyte adhesion to the vascular wall by interfering with the binding ability of leukocyte adhesion molecule CD11/CD18 to the endothelial cell surface [20]. Reactive oxygen species (ROS) and angiotensin II, as well as proatherogenic agents such proinflammatory cytokines, are all prevented by eNOS-derived NO from causing endothelial cells to die. The anti-inflammatory and anti-atherosclerotic properties of endothelium-derived NO may also be influenced by apoptosis inhibition. In addition, nitric oxide derived from eNOS can activate endothelial progenitor cells to play an important role in angiogenesis. NO produced in endothelial cells also has anti-atherogenic effects; the inhibition of platelet aggregation and adhesion protects smooth muscle from exposure to platelet-derived growth factors [21], which is destructive and produces erosion of the articular cartilage, eventually destroying the joint.

iNOS is not normally expressed in cells, but its expression can be induced by bacterial lipopolysaccharides, cytokines and other factors, and it can be stimulated in almost any cell or tissue. Once expressed, iNOS is continuously activated and not regulated by intracellular Ca^2+^ concentration. When induced in macrophages, inducible NOS produces large amounts of NO, which results in cytotoxicity [22]. Increased NO can inhibit important enzymes that have iron in their catalytic centers because of its affinity for protein-bound iron, including ribonucleotide reductase, which is involved in DNA replication; *cis*-aconitase; and iron-sulfur cluster dependent enzymes involved in mitochondrial electron transport [23]. Additionally, increased NO production in the stimulated macrophages has the potential to directly interfere with DNA of target cells, leading to DNA strand breaks and fragmentation. The interaction of these effects may underlie the cyto-inhibitory and cytotoxic effects produced by NO on parasitic microorganisms and certain tumor cells. However, when high levels of inducible NOS-derived NO produced by activated macrophages are released in the wrong location, normal cells may be damaged, leading to autoimmune disorders [24].

## 3. The Pathological Changes of RA

### Histological Changes

**Figure 2:**
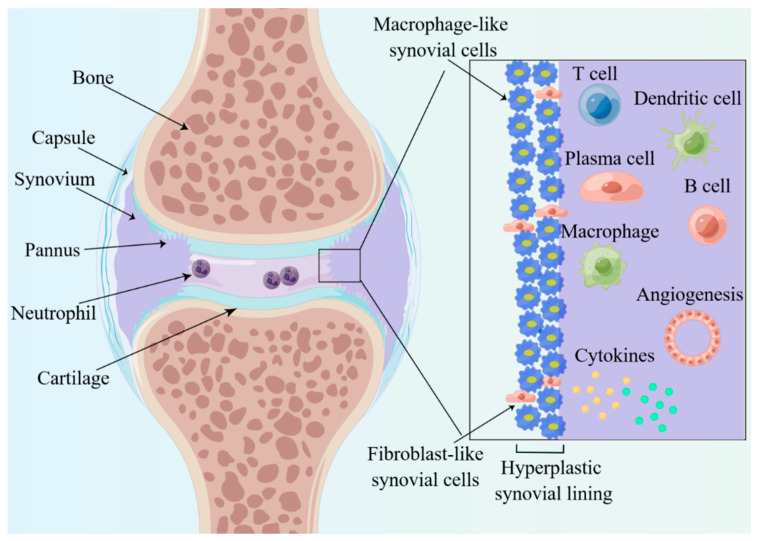
Histological features of inflamed joints of RA. The enlarged synovial lining layer consists of macrophage-like and fibroblast-like synovial cells and then forms many villous projections called pannus. Significant changes also take place, with a marked infiltration of cytokines, plasma cells, macrophages, neutrophils, B cells and T cells.

Synovitis is the key to the pathophysiology of RA, and synovial structure changes as the RA progresses. In the early stage of onset, tissue edema and fibrin deposition are evident, with clinical manifestations of joint swelling and pain [25]. The characteristic of this stage is the entry of leukocytes and neutrophils from the blood into the synovial tissues (Figure 2). In the short term, the synovial membrane becomes hypertrophic, usually forming 10 or more cells consisting of macrophage-like (type A) and fibroblast-like (type B) synovial cells (Figure 2), and forms many villous projections that protrude into the joint cavity or invade the cartilage and subchondral bone. The villous projections, also called pannus, damage and produce erosion of the articular cartilage, which is the distinctive pathological basis of destruction, deformity and dysfunctions of joints. Significant changes also take place in terms of the number and the sub-cellularity of immune cells, with a marked infiltration of single nucleated cells, such as plasma cells, macrophage, B cells and T cells. Moreover, there exist changes in the expression of cell surface adhesion molecules, proteases, protease inhibitors and many cytokines in synovitis.

## 4. The Pathogenesis of RA

The accurate pathogenesis of RA is unclear at present. Currently, genetic factors and epigenetic mechanisms could be not fully responsible for the whole process of RA development. Environmental factors, including smoking, diet, obesity, infections, microbiota and chronobiology changes in the production of certain hormones can increase the risk of RA in individuals who carry susceptibility genes [26]. The effect of the combination of these factors causes changes in NOS, leading to synovial cell proliferation and subsequent synovial opacity formation, cartilage damage and bone erosion. Therefore, there are many hypotheses about the cause of rheumatoid arthritis [26]. The development of RA is divided into three stages, characterized by autoimmune priming, tissue attack and chronic inflammation related to NOS/NO pathways.

### 4.1. RA and Autoimmunity

The tissue destruction caused by the autoimmune effects of RA manifests as synovitis, which is jointly initiated and maintained by the combination of the activation of various immune cells, including the different dendritic cells (DCs) subtypes, T-cells, macrophages, B-cells, neutrophils and other cells such as fibroblast and osteoclasts [27]. 

**Figure 3:**
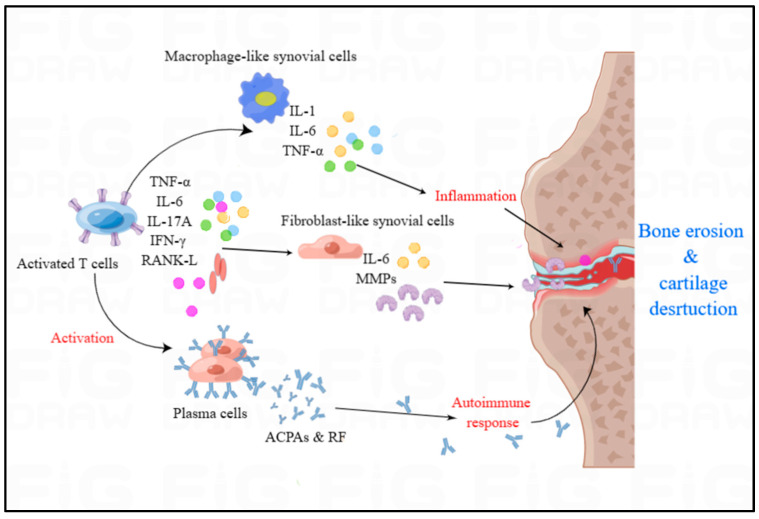
The overview of immune cells and cytokines in RA. Autoimmunity and inflammatory cytokines play significant role in the development of RA. Activated T cells predominantly generate TNF-α, IL-6, IL-17A, IFN-γ, and RANK-L, which can activate the plasma cells, fibroblast-like synovial cells and macrophage-like cells. Macrophage-like cells can produce IL-1, IL-6 and TNF-α to induce the inflammation in the synovium of RA. Activated plasma cells can release the autoantibodies including ACPAs and RF to trigger autoimmune responses in RA.

Accumulating evidence indicates that the concentration of DCs in the plasma of RA patients results from the recruitment of DCs in the flamed joints. This distribution and changed function of DCs contributes to the development of RA. Activated DCs are capable of producing IFN-α, IFN-β, IL-18 and IL-23, increasing the generation of autoantibodies by expression of anti-apoptotic B cell activating factor, and promoting the presentation of autoantigens to T cells, which potentiates the autoimmune response and maintains the inflammation. Activated T cells can secrete IL-2, IFN-γ and TNF-β and Th1 cells can activate other immune cells, such as macrophages and B cells. Th 17 cells, activated in synovial joints, are capable of recruiting neutrophils, activating immune cells and enhancing osteoclastogenesis [28]. Macrophages are activated and then generate pro-inflammatory cytokines including IL-1β, IL-6 and TNF-α, which can accumulate and activate other immune cells in the synovium [27]. Activated fibroblasts, together with other accumulated activated immune cells, promote osteoclastogenesis through the nuclear factor κB ligand receptor. In healthy people, there exist a balance between bone resorption by osteoclasts and bone formation by osteoblasts, while the balance is disrupted in RA patients, whose bone resorption becomes excessive. Increased expression of iNOS genes and proteins is seen under hypoxic conditions or oxidative stress, which promotes osteoclast differentiation [24]. The high concentration of RA specific antibodies, such as rheumatoid factor (RF) and anti-citrullinated protein antibodies (ACPAs), is associated with joint damage and high mortality (Figure 3), which there is no effective treatment to eliminate at present [27]. ACPAs are able to recognize the epitopes of the citrulline-containing protein and then bind to the RF, forming a complex which can cause the abundant complement activation to maintain the inflammation in the synovial capsule. The ACPAs themselves can be pathogenic by activating macrophages or by forming immune complexes and Fc receptor binding to activate osteoclasts [29], thereby aggravating bone destruction. RF can directly induce macrophage and cytokine activation, promoting the inflammation.

### 4.2. RA and Cytokines

Chronic inflammation and synovitis with joint swelling are the typical characteristics of RA [30]. Some studies have documented evidence for elevated cytokines concentrations in the affected tissues of pre-RA and RA patients compared with healthy subjects [31]. In RA, the concentration of pro-inflammatory cytokines is higher than anti-inflammatory cytokines, which can result in RA synovia [31]. Macrophage-like synoviocytes and fibroblast-like synoviocytes are the main sources of cytokines. Macrophage-like synoviocytes may result in the overproduction of proinflammatory cytokines including IL-1, IL-6 and TNF-α, thus promoting persistent inflammation and joint destruction (Figure 3). Fibroblast-like synoviocytes express IL-6, MMPs, prostaglandins and leukotrienes, which manifests an aggressive inflammatory, stromal regulatory and invasive phenotype in the synovial compartment. The inflammatory environment in the synovial compartment is induced and maintained by the cytokine and chemokine network, including tumor necrosis factor TNF-α, IL-6, IL-17A, interferon IFN-γ and receptor activator of nuclear factor κB ligand (RANK-L) (Figure 3), which can activate endothelial cells and accumulate immune cells in the synovial compartment to trigger and regulate inflammation via the binding of cytokines to cell receptors. Recently, some successful cellular RA models induced by TNF-α have induced fibroblast-like synoviocytes [32]. The current success of drugs blocking TNF-α as well as IL-17A receptors in the treatment of rheumatoid joints confirms that TNF-α and IL-17A mediated transmissions play a key role in rheumatoid arthritis [33].

One of the key inflammatory cascades is the overproduction and overexpression of TNF-α through activating T-, synovial macrophages, B-, and NK-cells, which may trigger synovial inflammation and bony erosions. The overproduction of TNF-α is caused by the interactions between T cells and B cells, as well as the interaction between fibroblasts and macrophages, and can induce the generation of IL-1β and IL-6, in which TNF-α plays a significant function by activating cytokine and chemokine expression, protecting synovial fibroblasts, generating endothelial cell adhesion molecules, promoting angiogenesis, inhibiting regulatory T cells and inducing pain. Recent paper reported that TNF-α promoted the secretion of RANK-L, which can interact with TNF-α by a RANK-L-independent mechanism to induce the formation of osteoclasts, further potentiating osteoclastogenesis [34,35]. IL-17A secreted by TH17 cells can also produce pro-inflammatory cytokines, promote the differentiation of mature osteoclasts and migration of endothelial cells, and increase the generation of RANK-L, MMP-1 and vascular endothelial growth factor (VEGF), which can cause angiogenesis, perpetuation of inflammation, bone erosion and cartilage destruction [36,37]. The high concentration of IFN-γ is indicated in the synovium, synovial fluid and plasma [38]. IFN-γ can promote antigen presentation and activate macrophages by the Janus-activated kinase-signal transducer and activator of transcription 1 (JAK–STAT1) pathway, as well as the mitogen activated protein (MAP) kinase-, phosphatidylinositol 3-kinase (PI3K)- and nuclear factor kappa-light-chain-enhancer of activated B cells (NF-κB)-pathways [39]. In addition to the local joint’s inflammation, peripheral inflammation and cytokine release can trigger changes in brain metabolism related to reduced cognitive function [40,41]. Current research had demonstrated that non-selective histone deacetylase (HDAC) inhibitors (HDACi) can suppress inflammatory gene expression in models of RA fibroblast-like synoviocytes in order to play anti-inflammatory role [42].

## 5. NOS/NO Pathway in RA Pathogenesis

NOS and NO are critical signal molecules with various physiological and pathological effects, which regulate a variety of cellular events. NO, produced by NOS, is an endogenous vasodilator and intercellular messenger in the cerebral and peripheral blood flow [43]. NO plays an important role in self-protective inflammatory and immune responses, but excessive endogenous NO may cause inflammatory diseases such as RA. There are several kinds of cells can generate NO, such as macrophages, fibroblasts, neutrophils, endothelial cells, osteoblasts and osteoclasts in the synovium [44]. It is reported that indications of NO-dependent tissue injury and overproduction of endogenous NO synthesis are present in RA, which suggests that overproduction of NO is important in RA [7]. Some studies showed that the symptoms of RA can be reduced by inhibiting the activity of NOS. The pathogenesis of RA involves a variety of mediators including NO, peroxide and cytokines such as IL-6, in connection with the main characteristics of RA. Although the pathogenesis of RA still remains unclear, NO and three isoenzymes of NOS are considered to play significant roles in the pathogenesis of RA.

### 5.1. NOS/NO Pathway Involved in RA Autoimmunity

NO plays an important role in the immune system, and can be produced by many different immune cell types, including dendritic cells, NK cells, macrophages, mast cells, eosinophils and neutrophils. NO and these immune cells are involved in the development of RA. 

T cell functions are considered to have a key role in the pathogenesis of RA. NO has been proven to regulate T cell functions in normal ranges, but excessive NO can result in T cell dysfunction. In mammals, NO regulates T cell activation, mitochondrial membrane potential and mitochondrial biogenesis. Based on the current reports, T helper 1 (Th 1)/Th 2 and Th 17/Treg imbalance can potentiate the activity of RA. Many studies show that eNOS-derived NO is able to enhance the signal transduction of antigens to T cell receptor at the immunological synapse, promoting the activation of T cells [45]. After activation, CD4 T cells proliferate and differentiate into Th 1 and Th 2 cells. Th 2 cells express various anti-inflammatory cytokines with protective effects for humans. NO can selectively promote the proliferation of Th 1 and influence the Th 1/Th 2 balance, which is essential in chronic inflammatory disease. Moreover, NO also increases the amount of Th 17 cells mediating the activation of T cells, which is associated with the onset of RA and the destruction of bone and cartilage of inflamed joints [46]. Down-regulated Treg cells have been demonstrated to increase the humoral and cellular immune response and deteriorate RA condition in CIA model mice [47]. Overproduction of NO regulates mitochondrial membrane potential in human T cells to induce mitochondrial hyperpolarization related to depletion of ATP, which then causes T cells necrosis. T cells necrotic materials activate monocytes and dendritic cells [48] which act as the antigen-presenting cells, in turn activating the T cells to form the vicious circle enhancing the RA autoimmunity.

Macrophages also play a significant role in human immune responses; they can participate in the immune defense system against pathogens and microorganisms and act as antigen-presenting cells. Plenty of activated infiltrated macrophages can generated excessive NO in an inflamed synovium [49]. Overproduction of NO can induce synovial angiogenesis and hyperplasia and promote synovial lesions.

### 5.2. NOS/NO Pathway Involved in RA Oxidative Stress

More and more evidences suggest that oxidative stress can accelerate the development of RA. Oxidative stress markers, NO and peroxides (PO) also constitute the major pathophysiological factors of RA. NO can react with superoxide (O_2_*^−^*) to produce peroxynitrite (ONOO^−^), affecting the cytoplasmic redox balance. The excessive accumulation of free radicals could trigger oxidative stress, which contributes to the pathogenesis of RA [50]. Reactive oxygen synthase (ROS) and reactive nitrogen synthase (RNS) can participate in a variety of cellular activities.

The excessive accumulation of harmful free radicals and superoxide can trigger an imbalance of oxidants and antioxidants, resulting in oxidative stress. ROS generate oxygen in an oxidative stress environment. The reaction rate of O_2_^−^ with NO is 3 to 4 times faster than that of superoxide dismutase. Therefore, if the O_2_^−^ concentration increases or NO levels rise excessively, they will react to produce the massively cytotoxic substance ONOO^−^ [51]. ONOO^−^ can cause joint cell degradation, including lipid peroxidation, protein dysfunction, and DNA damage, thus exacerbating the condition of RA [20]. Oxidative stress can decrease NO levels and NO bioavailability by producing ONOO^−^, thus further exacerbating the oxidative stress condition and finally forming a vicious circle. One recent paper demonstrated that iNOS promote the release of ONOO^−^. According to Costa et al., nitric oxide metabolites and total free radical capture antioxidant parameters are closely related with RA pathogenesis. The findings demonstrate that the inhibition of iNOS is considered to be a potential therapeutic direction for the treatment of RA [50]. Moreover, a condition called eNOS uncoupling exists in RA patients, wherein eNOS reacts with oxygen to produce O_2_*^−^* instead of NO due to insufficient substrates or cofactors [52]. Thus, the overproduction of ONOO^−^ may occur. Furthermore, ONOO^−^ can cause cytotoxicity and dysfunction of NO, eventually reducing the bioavailability of NO.

### 5.3. NOS/NO Pathway Involved in RA Inflammatory Cascade

**Figure 4:**
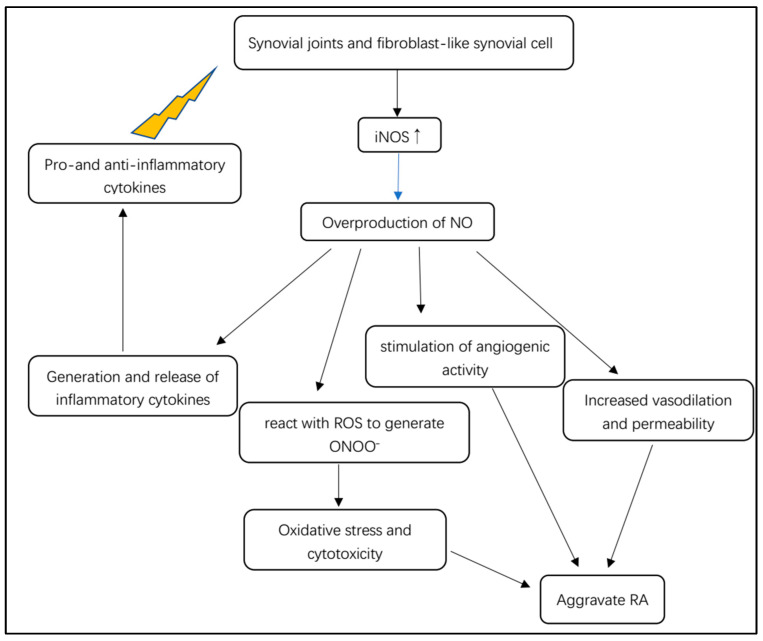
Overview of the pathological mechanism of iNOS-dependent NO for RA.

In rheumatoid arthritis, NO is involved in inflammation, angiogenesis and tissue degradation (Figure 4). Pro- and anti-inflammatory cytokines stimulate synovial macrophages and fibroblast-like synovial cells to express iNOS (Figure 4) [31]. iNOS stimulated by cytokines is responsible for the local overproduction of NO in joints affected by RA [31]. iNOS is considered to be mainly expressed in unhealthy states, such as inflammatory microenvironments, where it can be activated or triggered by various cytokines such as TNF-α, TNF-β and IL-6. Furthermore, cell senescence can express a special pro-inflammatory phenotype, indicating that senescent synoviocytes can secrete inflammatory factors such as TNF-α, TNF-β and COX-2 [53]. After immunological or microbial stimulation, iNOS can be induced to remain active to produce excessive NO, mediating the generation and release of inflammatory cytokines [22]. Other pro-inflammatory functions of NO in arthritis can promote vasodilation and permeability of TNF and IL-1 release by leukocytes and initiate angiogenic activity via monocytes or macrophages [54]. The overproduction of iNOS-derived NO can cause oxidative damage to the macromolecular substances of a cells, such as nucleic acid, lipids and proteins, thereby damaging mitochondrial functions and thus disturbing the normal cellular events [13]. Bioenergy deficiency may promote inflammatory cascade and aggravate synoviocytes dysfunction.

Complex interactions between inflammatory mediators such as TNF-α, IL-1β, COX-2 and NO are responsible for articular cartilage destruction. Arthritis promotes inflammatory responses in rats through the activation of IκB kinase (IKK), ubiquitination/protein vesicle degradation of IκB and the transport of activated NF-κB from the cytoplasm to the nucleus, which activates the transcription factor NF-κB. Activated NF-κB in the nucleus binds to the promoter region of target genes and upregulates the production of pro-inflammatory cytokines, iNOS and other inflammatory mediators, leading to cartilage destruction [50]. The increased NO concentration and oxidative stress can induce inflammation, which can activate the TLR4/NF-κB pathway to produce more inflammatory mediators. These inflammatory mediators can in turn directly trigger the over-expression of iNOS and excessive NO production. These events form a vicious circle, exacerbating the condition of RA patients. Nest disruption protein 2 (DVL2) is highly expressed in the articular cartilage of RA patients and participates in the destruction of RA articular cartilage by promoting chondrocyte destruction-related gene expression through activation of the WNT/β-catenin pathway. DVL2 overexpression upregulates COX-2, iNOS, matrix metalloproteinase MMP-2, MMP-3 and MMP-9 mRNA expression, and when TNF-α stimulation was added, COX-2, iNOS, MMP-2, MMP-3 and MMP-9 mRNA expression was more pronounced [55]. Inflammation induces the exudation of plasma proteins which can cause tissue edema, resulting in swelling joints. In RA, chronic inflammatory arthritis can lead to NOS-dependent draining lymphatic vessel dysfunction. Due to the decreased outflow of inflammatory cells and soluble factors from the affected joints, the contractility of lymphatic vessels decreases or the lymphatic vessels collapse, thereby exacerbating the disease and possibly triggering arthritic episodes due to decreased outflow of inflammatory cells and soluble factors from the affected joints [18].

### 5.4. NOS/NO Pathway Involved in RA Circadian Rhythm

The progression of RA is closely linked to the chronobiology changes in the production of certain hormones and inflammatory mediators that affect the course of the disease and the outcome of treatment. RA is perceived as a disease disrupting the circadian rhythm of steroid hormone or cytokine production. In recent years, circadian rhythm disturbance of the inflammatory process in RA has been recognized as one of the important inflammatory mechanisms that triggers inflammation, destruction and proliferation of synovial joints [56]. eNOS and soluble Toll-like receptor 2 (sTLR2) are involved in the regulation of angiogenesis, osteoclast-genesis and immune response [57]. The main pathogenesis of RA is controlled by the biological clock gene angiogenesis [25]. Further studies on the circadian rhythm of angiogenic mediator production in RA patients may be important and relevant. Angiogenesis plays an important role in RA, but the circadian rhythms of angiogenic mediator production, particularly eNOS, which is involved in the regulation of endothelial function, inflammation and bone reconstitution processes, are not known. Circadian variations in circulating eNOS levels exist in female RA patients and healthy women. In contrast, in RA patients, decreased eNOS production, especially in the morning, means elevated activity of RA. Therefore, the circadian rhythm of circulating eNOS may be opposite to the circadian rhythm of major inflammatory regulators production in RA [56].

### 5.5. NOS/NO Pathway Involved in RA Complications

#### 5.5.1. The Effect of NOS/NO Pathway in Cardiovascular Manifestation of RA

**Figure 5:**
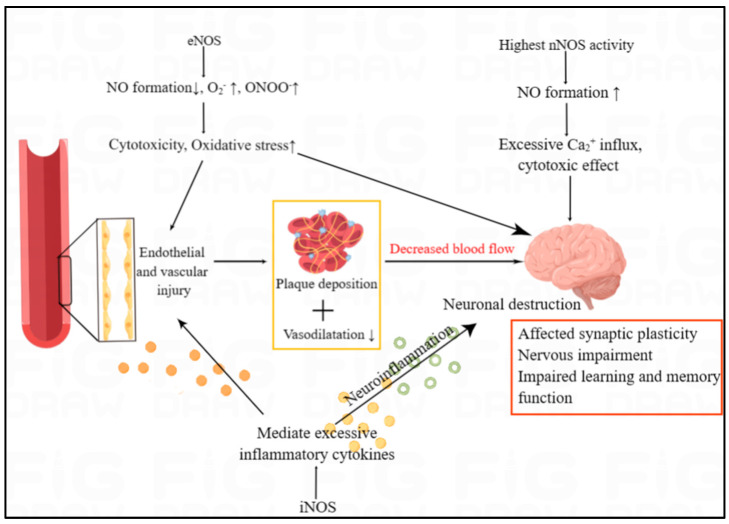
The overview of NOS/NO in the cardiovascular and neurological manifestation of RA. The three NOS isoforms, with entirely different functions, jointly influence brain function. In humans, nNOS activity is highest in the brain, affecting the synaptic plasticity. In both the endothelium and the brain, iNOS and eNOS can trigger inflammatory responses and high oxidative stress, which can lead to endothelial injury and neuronal destruction. Endothelial and vascular injury are associated with plaque deposition and decreased vasodilation, which results in the decreased blood flow to brain.

Cardiovascular disease (CVD) is regarded as a common complication in RA patients. RA increases the risk of CVD, which accounts for the highest morbidity and mortality observed in RA patients. Endothelial dysfunction resulting from impaired nitric oxide synthesis increases the risk of the cardiovascular disease. eNOS uncoupling increases cardiovascular mortality associated with autoimmune rheumatic diseases. Oxidative stress has a significant role in endothelial dysfunction, which is also identified to be caused by eNOS uncoupling (Figure 5). The conversion of uncoupled eNOS into superoxide-producing enzyme can decrease the production level of NO and enhance pre-existing oxidative stress, which plays a critical role in the process of atherosclerosis [58]. 

Vascular dysfunction and abnormal morphology may cause aggravate atherosclerotic condition. It is considered to be one of the main drivers of CVD in RA patients, while systemic inflammation plays an important role in the initiation of endothelial and vascular injury. Recent reports have shown that NOS inhibitors asymmetric dimethylarginine (ADMA) and symmetric dimethylarginine (SDMA) have emerged as new CVD risk factor determinants. ADMA was found to be an indicator of CVD risk in RA patients through meta-analysis [59]. In RA, several studies have shown that abnormalities in dimethylarginine metabolism can be attributed to multiple factors, such as systemic inflammation, reduced degradation or enhanced synthesis [60]. The close association between inflammation, dimethylarginine and endothelial dysfunction suggests that identifying dimethylarginine regulators may help demonstrate the process of atherosclerosis in RA. The alanine-glyoxylate transaminase 2 (AGTX2)-dependent pathway is a relatively new dimethylarginine catabolic pathway, and variants in the AGTX-2 gene may affect dimethylarginine levels in RA patients, leading to elevated CVD risk and endothelial dysfunction of dimethylarginine in RA-related atherosclerotic disease [61]. RA is a chronic disabling autoimmune disease in which premature atherosclerosis is a major symptom beyond joint manifestations. The mechanisms that accelerate atherosclerosis in rheumatoid arthritis disease remain unclear to date. NO, the endogenous messenger of endothelial cell synthesis, is thought to be a key part in the pathogenesis of RA atherosclerosis, thereby altering local homeostatic mechanisms and favoring vascular injury and plaque deposition.

#### 5.5.2. The Effect of NOS/NO Pathway in Neurological Manifestations of RA

Brain lesion is rarely reported in RA, but cognitive decline was studied in the article of Pankowski et al. [62]. Neurological manifestations of RA range from mild hand sensory abnormalities and carpal tunnel syndrome to cognitive dysfunction [63,64]. Compressive neuropathy, particularly carpal tunnel syndrome, is the most common abnormality of the peripheral nervous system in RA [63]. The three NOS isoforms, with entirely different functions, jointly influence brain function. It has been reported that the reduction of eNOS activity as well as the overactivation of nNOS and iNOS can lead to brain lesion. Several studies have shown that NO has both neuroprotective and neurotoxic effects, which depends on its bioavailability, concentration and the redox state of the microenvironment of the body [65]. nNOS is abundant in central and peripheral neurons, and nNOS-derived NO play an important role in learning and memory ability via participating in changing the concentration of Ca^2+^. Ca^2+^ has a significant role in affecting synaptic plasticity [66]. iNOS can mediate excessive release of inflammatory cytokines which can leads to neuroinflammation, thus continuously resulting in neuronal destruction (Figure 5). eNOS produces nitric oxide that plays a critical role in maintaining vascular function and antithrombotic effects. Low eNOS activity is associated with stroke and neurovascular dysfunction. Results from Tan XL et al.’s study provides evidence that partial eNOS deficiency causes spontaneous cerebral thrombosis, progressive cerebral amyloid angiography and cognitive damage [67].

## 6. Disease-Modifying Antirheumatic Drugs Targeting NOS/NO Pathway

Excessive and persistent inflammatory responses are a feature of RA. Therefore, the key to preventing and treating RA lies in managing inflammation and studying anti-inflammatory therapies. Several of the most promising strategies to design therapeutic compounds for RA includes targeted suppression of iNOS [68]. NF-κB, as an upstream signal of iNOS, modulates the expression of a range of cytokines, chemokine and inflammatory mediator genes in activated macrophages. Thus, NF-κb has emerged as a promising drug target for reducing inflammation. All these above provide ideas for the development of disease-modifying antirheumatic drugs (DMARDs) and the discovery of pharmacological mechanisms is significant for applications of drugs (Table 1). DMARDs refers to medicines that interfere with signs and symptoms of RA, inhibiting progression of joint damage. 

### 6.1. Conventional DMARDs

In recent years, with the in-depth research on some conventional drugs for the treatment of RA, the specific mechanisms of action and therapeutic effects have been explored. The elucidation of the mechanism of DMARDs will help to control adverse reactions. For example, Etanercept can significantly alleviate arthritis, which may be achieved by increasing eNOS phosphorylation and expression, decreasing arginase-2 and subunit of NOX p22-phox expression and decreasing O_2_^−^ production. In addition, Etanercept has been found to exert pleiotropic effects on endothelial pathways, improving endothelial dysfunction and reducing risk factors of cardiovascular disease, with significant increases in systolic blood pressure and heart rate [69]. Indometacin (IDMT) is an analgesic used to relieve pain in RA. IDMT was found to be able to alleviate RA-induced endothelial cell damage through two different pathways, higher concentrations of IDMT through the IKK-COX-2/TNF-α pathway, and lower concentrations through the PPARγ-Akt-eNOS pathway. Furthermore, the mechanism of another conventional antiarthritic drug, Auranofin (AF), has been elucidated. AF is an anti-inflammatory gold compound that has been used to treat RA for more than four decades. AF can inhibit the upregulation of NOX-4 and the translocation of NF-κB in RA macrophages by binding to Toll-like receptors. AF can suppress expression levels of inflammatory stimulants iNOS and reduce NO levels through the induction of pro-inflammatory proteins and mRNA by the upstream regulatory factor NF-κB [70].

Furthermore, several drugs not previously used for the treatment of RA have also been found to have therapeutic effects on the condition, and the pharmacological mechanisms of these drugs have also been explored. Allopurinol, a classic inhibitor of uric acid production, can significantly inhibit the NF-κB pathways and achieve RA therapeutic effects by regulating the activated iNOS/NO axes [71]. Antianginal drug Nicodil was also proven to have a protective effect for RA by correcting iNOS and eNOS levels in vitro model [72]. In recent years, the calcium channel blocker Verapamil has been reported to have the ability to reduce inflammation and joint destruction in RA. Verapamil can reduce the expression of inflammatory cytokines such as iNOS via inhibiting the activation of the NF-κB signaling pathway [73]. Aliskiren and tadalafil, as potential anti-rheumatic drugs, can reduce the expression of iNOS and enhance the expression of eNOS. Thus, oxidative stress and NO content are decreased in joint tissues. It is worth mentioning that the therapeutic effect of the renin inhibitor Aliskiren is particularly close to the gold standard conventional antirheumatic drug methotrexate [74]. Drugs such as the P2Y12 receptor blocker Ticagrelor, the modulator of selective estrogen receptor Tamoxifen and the inhibitor of sodium/glucose transporter-2 Empagliflozin can significantly increase the levels of eNOS in aortic tissues and correct RA-induced vascular responsiveness defects [75]. Roscovitine, a Cdk5 inhibitor as well as an anticancer agent, has recently been shown to provide a strong suppression of iNOS expression and reduce NO production in response to inflammatory stimulation when combined with GC therapy, providing a replacement of high-dose GC therapy to reduce side effects [76]. Edaravone can reduce the concentration of eNOS, heme oxygenase-1, perisynovial ROS production and inflammatory factors. Edaravone has been shown to reduce the expression of NF-κB p65 by scavenging ROS and alleviates tissue damage and arthritis symptoms [77]. The phosphodiesterase (PDE) inhibitors Vardenafil and cilostazol can induce vasodilation and maintain endothelial integrity. Vardenafil and cilostazol can up-regulate vascular eNOS to exhibit protective effects against vascular reactivity defects and endothelial dysfunction in RA patients [78]. All-trans retinoic acid (ATRA) is a vitamin A derivative with various immunomodulatory effects through inhibiting NF-κB translocation and iNOS expression in peripheral blood mononuclear cells [79]. 

### 6.2. Novel DMARDs 

New synthetic anti-RA drugs mostly achieve anti-inflammatory effects by targeting specific receptors. An active proinflammatory mediator with several functions, macrophage migration inhibitory factor (MIF), contributes to the development of RA. MIF inhibitor compound 3-[(biphenyl-4-ylcarbonyl) carbamothioyl] amino benzoic acid (Z-590) has potent anti-arthritis effects achieved by inhibiting macrophage activation and significantly improving joint inflammation and articular cartilage damage. Z-590 can significantly inhibit the production of NO through suppressing expression of iNOS in RA macrophages in and in vitro model [80]. By activating NO/cGMP/ATP-sensitive potassium channels without impairing inflammatory processes, the 7-nicotinic acetylcholine receptor (7nachRs) agonist choline can control inflammatory hyperalgesia, suggesting its potential therapeutic potential for the treatment of RA pain [81]. Cannabinoid receptor 2 (CB2) has powerful anti-inflammatory effects, and a selective agonist of CB2, 4-quinolone-3-carboxamides (4Q3C), can significantly improve arthritis and reduce bone erosion. Treatment with 4Q3C reduced the expression of iNOS [82]. Soluble epoxide hydrolase (sEH) can rapidly convert endogenous anti-inflammatory compounds, for example, epoxyeicosatrienoic acids, into less active forms. Thus, inhibition of sEH enzymes is a potent strategy to reduce nociception and inflammation. SEH enzyme inhibitor 1-trifluoromethoxyphenyl-3-(1-propionylpiperidin-4-yl) urea (TPPU) can attenuate arthritic hyperalgesia and inflammation via peripheral intervention. TPPU significantly reduced iNOS protein expression and local concentrations of proinflammatory cytokines [83]. 2-Deoxyglucose (2-DG), a glycolysis inhibitor, was shown to have anti-arthritic properties and can reduce cell infiltration, synovial hyperplasia and bone erosion in RA patients. Its therapeutic mechanism has recently been found to be related to repression of NF-κB in peritoneal macrophages in rat model of RA, thereby promoting the transformation of macrophages type from M1 to M2. By regulating macrophage polarization as well as metabolic changes, 2-DG increases arginase 1 (Arg1) levels and decreases iNOS expression [84].

Coupling active structural motifs to synthetic or natural molecules is an alternative strategy to develop novel drugs. Through different substituted coumarin moieties coupled to natural phenols via amide bonds, an immunosuppressant, YR2e, was synthesized with dual activity to inhibit iNOS and NBT reduction. The compound YR2e can be used as a drug to prevent RA [85]. Chalcone derivatives, such as methoxyphenyl-and coumarin-based derivatives, have inhibitory effects on NO production. These chalcone derivatives all produce anti-inflammatory effects by reducing the expression of NF-κB and phosphorylated Iκb in LPS-stimulated macrophages [86]. In addition, a new potent anti-RA agent, 3,4,5-trimethoxystyryl-1 H-pyrazolo (4,3-D) pyrimidine, was designed based on the preliminary structure of pyrazolo (4,3-D) pyrimidine compounds. It has a lower toxicity but stronger inhibitory ability for NO release. It can interfere with the stabilization and formation of the active dimer iNOS [87]. Another example is the tylophorine-based compounds DBQ 33B (2-Ethyl-7,10,11-trimethoxy-1,2,3,4-Tetrahydrodibenzo [f,h]-isoquinolin-4-ol), which can down-regulate expression of iNOS and significantly improve the severity and morbidity of RA [88].

In recent years, with the rise of nanotechnology, novel methods for developing synthetic RA drugs have gradually emerged. Treatment of RA with Retinoic acid-platinum (II) Complex (RT-Pt (II)) can act by targeting the NF-κB pathway to inhibit the mRNA and protein expression of iNOS in RA models in rats. This nano complex RT-Pt (II) can significantly reduce the inflammatory response of RA synoviocytes in an RA model [89]. Gold nanoparticles (AuNGs) also have anti-arthritic activity by reducing inflammatory mediators such as iNOS and NF-κB and correcting oxidative stress, thereby reducing inflammation and bone erosion in the RA model [90]. Furthermore, Rutin conjugated gold nanoparticles (R-AuNPs) can down-regulate the expression of NF-κB and iNOS and significantly reduce NO levels in the treatment of RA. In addition, core–shell nanocarriers can achieve a slow and sustained release of drugs, exhibiting a stronger ability to inhibit inflammatory mediators than free drugs, thus achieving a better therapeutic effect on RA. Glycyrrhizic acid encapsulated by amino cellulose-grafted polycaprolactone (PCL-AC) with a budesonide coating can reduce inflammatory markers such as iNOS with good effect to reduce the symptoms of swelling and erythema in RA [91]. Another NP example is breathing micelle (BM), a new type of artificial respiration material capable of gas exchange, which is able to inhale NO and exhale CO. BM can simultaneously eliminate excessive NO production and reduce proinflammatory cytokines in RA macrophages in an in vitro model. Studies have shown that the efficacy of BM in the treatment of RA is even better than that of traditional NSAIDs such as dexamethasone in certain circumstances, possibly due to the combined effects of NO depletion, CO-mediated inactivation of iNOS and heme oxygenase-1 (HO-1) activation [92]. Another material similar to BM is nitric oxide-scavenging nanogel (NO-SCV gel), which also shows a better effect of inhibiting the onset of RA than dexamethasone. NO-SCV gel was prepared by adding NO cleavable cross-linking agent (NOCCL) followed by polymerization with acrylamide solution. When NO-SCV gels are exposed to NO, NOCCL is readily cleaved by consuming and scavenging NO molecules, thus repressing inflammation [93].

## 7. Conclusions

This review attempts to evaluate role of nitric oxide and nitric oxide synthases in rheumatoid arthritis. We summarize the pathogenic mechanisms of RA found in recent years, the physiological function of the nitric oxide/nitric oxide synthases and how their abnormality accelerates the course of rheumatoid arthritis, as well as the latest research on drugs and treatment for RA. Overproduction of NO can potentiate the autoimmune response, promote the inflammation of synovium and increase the oxide stress, which results in bone erosion and cartilage destruction. The high levels of NO detected in plasma and synovial fluid of RA patients also contributes to the complications, such as cardiovascular and neurological manifestations. Based on the above review, it is clear that NOS/NO, with their upstream and downstream signaling pathways, have a significant role in pathogenesis of RA. Targeting NOS can be an effective method to control inflammation in RA and its complications. In addition, research on medicines for RA in recent years is summarized, and pharmacological mechanism of many conventional and novel synthetic drugs are proved, in order to provide guidance for drugs administration.

## Figures and Tables

**Table 1:** Synthetic and Natural DMARDs based on NOS/NO pathway.

Conventional		Novel	
Name	Target	Name	Target
Etanercept	arginase-2	MIF inhibitor compound (Z-590)	iNOS
Auranofin	Toll-like receptors	α7nachRs agonist (choline)	NO/cGMP/ATP-sensitive potassium channels
Allopurinol	NF-κB and iNOS	CB2 agonist (4Q3C)	iNOS
Indometacin	PPARγ-Akt-eNOS pathway	sEH enzyme inhibitor (TPPU)	iNOS
Nicocdil	iNOS and eNOS	glycolysis inhibitor (2-deoxyglucose)	NF-κB and iNOS
Aliskiren, tadalafil	NF-κB	coumarin moieties coupled to phenols (YR2e)	iNOS
Ticagrelor, Empagliflozin, Tamoxifen	eNOS	chalcone derivatives	NF-κB and phosphorylated Iκb
Roscovitine	iNOS	pyrimidine-based compounds tylophorine-based compounds	iNOS
Edaravone	NF-κB p65	RT-Pt (II)	NF-κB and iNOS
Verapamil	NF-κB signaling pathway	AuNGs R-AuNPs	NF-κB and iNOS
All-trans retinoic acid	NF-κB and iNOS	PCL-AC	iNOS
Vardenafil and cilostazol	eNOS	BM NO-SCV gel	iNOS HO-1

## Data Availability

Not applicable.
